# Ethical Education

**Published:** 1899-06

**Authors:** 


### ETHICAL EDUCATION.

That every veterinary college and school of this country needs to look after the ethical training of its graduates, lest they so far depart from the conservative lines marked out for all true professional men, is strongly evinced by the publication in this issue of an advertising letter of a recent graduate in a Minnesota paper. Such a lack of ethical training must tell forcibly against any school when painfully brought to public notice among an intelligent community by one of her graduates, not to speak of the adverse opinion created in a measure against the whole profession. Will any one at this day deny the value of higher veterinary education? Will any one question the need of State laws that shall debar such graduate from the practice of his profession? Surely such dangerous education must awaken the urgent need of higher authoritative boards than charters give to establish colleges.

